# Formate hydrogen lyase mediates stationary-phase deacidification and increases survival during sugar fermentation in acetoin-producing enterobacteria

**DOI:** 10.3389/fmicb.2015.00150

**Published:** 2015-02-25

**Authors:** Bram Vivijs, Leticia U. Haberbeck, Victor Baiye Mfortaw Mbong, Kristel Bernaerts, Annemie H. Geeraerd, Abram Aertsen, Chris W. Michiels

**Affiliations:** ^1^Laboratory of Food Microbiology and Leuven Food Science and Nutrition Research Centre, Department of Microbial and Molecular Systems, Faculty of Bioscience Engineering, KU LeuvenLeuven, Belgium; ^2^Division of Mechatronics, Biostatistics and Sensors, Department of Biosystems, Faculty of Bioscience Engineering, KU LeuvenLeuven, Belgium; ^3^Chemical and Biochemical Process Technology and Control Section, Department of Chemical Engineering, Faculty of Engineering ScienceKU Leuven, Leuven, Belgium

**Keywords:** mixed-acid fermentation, 2, 3-butanediol fermentation, acid stress, hydrogenase 3, formate hydrogen lyase

## Abstract

Two fermentation types exist in the *Enterobacteriaceae* family. Mixed-acid fermenters produce substantial amounts of lactate, formate, acetate, and succinate, resulting in lethal medium acidification. On the other hand, 2,3-butanediol fermenters switch to the production of the neutral compounds acetoin and 2,3-butanediol and even deacidify the environment after an initial acidification phase, thereby avoiding cell death. We equipped three mixed-acid fermenters (*Salmonella* Typhimurium, *S*. Enteritidis and *Shigella flexneri*) with the acetoin pathway from *Serratia plymuthica* to investigate the mechanisms of deacidification. Acetoin production caused attenuated acidification during exponential growth in all three bacteria, but stationary-phase deacidification was only observed in *Escherichia coli* and *Salmonella*, suggesting that it was not due to the consumption of protons accompanying acetoin production. To identify the mechanism, 34 transposon mutants of acetoin-producing *E. coli* that no longer deacidified the culture medium were isolated. The mutations mapped to 16 genes, all involved in formate metabolism. Formate is an end product of mixed-acid fermentation that can be converted to H_2_ and CO_2_ by the formate hydrogen lyase (FHL) complex, a reaction that consumes protons and thus can explain medium deacidification. When *hycE*, encoding the large subunit of hydrogenase 3 that is part of the FHL complex, was deleted in acetoin-producing *E. coli,* deacidification capacity was lost. Metabolite analysis in *E. coli* showed that introduction of the acetoin pathway reduced lactate and acetate production, but increased glucose consumption and formate and ethanol production. Analysis of a *hycE* mutant in *S. plymuthica* confirmed that medium deacidification in this organism is also mediated by FHL. These findings improve our understanding of the physiology and function of fermentation pathways in *Enterobacteriaceae*.

## INTRODUCTION

Within the *Enterobacteriaceae* family, a distinction is made between mixed-acid (e.g., *Escherichia*, *Salmonella,* and *Shigella*) and 2,3-butanediol fermenters (e.g., *Klebsiella*, *Serratia*, and *Enterobacter*) based on their fermentation end products produced during sugar fermentation. Mixed-acid fermenters ferment sugars to ethanol and a range of organic acids, including lactate, succinate, acetate, and formate. Formate can be further converted to H_2_ and CO_2_ by the formate hydrogen lyase (FHL) complex (White, 2000). Mixed-acid fermentation generally leads to rapid and strong medium acidification and even cell death. On the other hand, 2,3-butanediol fermenters use the mixed-acids pathway only during the early growth phase, and switch in the late exponential phase to a different fermentation pathway, in which pyruvate is converted to the neutral end products acetoin or 2,3-butanediol, thereby preventing excessive acidification (Van Houdt et al., 2006; Xiao and Xu, 2007). Moreover, after the initial decline of medium pH, 2,3-butanediol fermenters typically deacidify the medium toward more neutral values during stationary phase (Johansen et al., 1975; Yoon and Mekalanos, 2006; Van Houdt et al., 2007; Moons et al., 2011). This is in contrast to mixed-acid fermenters or 2,3-butanediol fermenters with an inactivated 2,3-butanediol pathway, where a sustained pH decrease is usually observed during sugar fermentation (Yoon and Mekalanos, 2006; Moons et al., 2011). Thus, 2,3-butanediol fermentation is apparently associated with stationary-phase deacidification. Synthesis of 2,3-butanediol from pyruvate requires three steps. First, the conversion of two molecules of pyruvate to α-acetolactate is catalyzed by the α-acetolactate synthase (α-ALS). Next, α-acetolactate is decarboxylated to acetoin by the α-acetolactate decarboxylase (α-ALD). In a last step, acetoin is reduced to 2,3-butanediol by the 2,3-butanediol dehydrogenase (BDH), which can also catalyze the reversed reaction. Each of these three reactions consumes an intracellular proton, and this potentially explains the observed stationary-phase deacidification. In *Serratia plymuthica* RVH1, a strain previously isolated from a food processing environment (Van Houdt et al., 2005), α-ALS and α-ALD are encoded by the *budB* and *budA* genes, respectively, which are located on the *budAB* operon (Moons et al., 2011). We previously showed that transfer of the *S. plymuthica* RVH1 *budAB* operon conveys to *Escherichia coli* the capacity to produce acetoin, to prevent lethal medium acidification and to reverse acidification (Vivijs et al., 2014a). In the present study, we transferred the *budAB* operon to some additional mixed-acid fermenting enterobacteria, *Salmonella* Typhimurium, *Salmonella* Enteritidis, and *Shigella flexneri*, and show that these also acquire the capacity to produce acetoin. However, acetoin production was not associated with stationary-phase deacidification in *S. flexneri*. This observation is remarkable since *Shigella* and *E. coli* are considered as a single species based on DNA homology (Fukushima et al., 2002). Thus, our results suggested the involvement of a deacidification mechanism different from proton consumption during acetoin production. To identify this mechanism, we performed random transposon mutagenesis in *budAB*-containing *E. coli* searching for mutants that lost their stationary-phase deacidification capacity but still produced acetoin. This led us to identify the FHL complex as the primary deacidification mechanism in 2,3-butanediol-fermenting *Enterobacteriaceae*.

## MATERIALS AND METHODS

### BACTERIAL STRAINS, PLASMIDS, OLIGONUCLEOTIDES, AND GROWTH CONDITIONS

The bacterial strains and plasmids used in this study are listed in **Table 1**. All bacteria were cultured in lysogeny broth (LB; 10 g/l tryptone, 5 g/l yeast extract, 5 g/l NaCl) or on LB agar (15 g/l agar) at 37°C except *Serratia plymuthica*, which was grown at 30°C. Media were supplemented with the following chemicals (Applichem, Darmstadt, Germany) when appropriate: 5 g/l glucose; 100 μg/ml ampicillin (Ap); 200 μg/ml carbenicillin (Cb); 30 μg/ml chloramphenicol (Cm); 5 μg/ml gentamicin (Gm); 10 μg/ml tetracycline (Tc); 50 μg/ml kanamycin (Km); and 1 mM isopropyl-β-D-thiogalactopyranoside (IPTG). Plasmids pTrc99A and pTrc99A-P_trc_-*budAB* were introduced into the mixed-acid fermenters by electroporation. All oligonucleotides used in this work are listed in **Table 2**, and were purchased from IDT (Haasrode, Belgium).

**Table 1:** Strains and plasmids used in this study.

Strain or plasmid	Relevant features	Reference
**Strains**
*Escherichia coli*
S17-1 λpir	*pro thi recA hsdR*^-^ *hsdM^+^* RP4: 2-Tc:Mu: Km Tn7 λ*pir*	Simon et al. (1983)
DH5α	F^-^ *endA1 hsdR17* (r_k_^-^, m_k_^+^) *supE44 thi-1*λ^-^ *recA1 gyrA96 relA1 deoR* Δ(*lacZYA*-*argF*)U169 Φ80d *lacZ*ΔM15	Grant et al. (1990)
MG1655	F^-^ λ^-^ *rph-1*	Guyer et al. (1981)
MG1655 *hycE*	Δ*hycE*	This study
*Salmonella enterica*
Typhimurium LT2	Wild-type	McClelland et al. (2001)
Enteritidis ATCC 13076	Wild-type	Tindall et al. (2005)
*Shigella flexneri*
ATCC 12022	Wild-type; serotype 2b	Daligault et al. (2014)
*Serratia plymuthica*
RVH1	Wild-type; biofilm isolate from food processing plant	Van Houdt et al. (2005)
RVH1 *budAB*	Δ*budAB::cat*, Cm^R^	Vivijs et al. (2014b)
RVH1 *hycE*	Δ*hycE*	This study
**Plasmids**
pTrc99A	Cloning vector carrying IPTG-inducible *trc* promoter (P_trc_); Ap^R^	Amann et al. (1988)
pTrc99A-P_trc_-*budAB*	pTrc99A carrying the *S. plymuthica* RVH1 *budAB* operon downstream of P_trc_; Ap^R^	Moons et al. (2011)
pKD3	Template plasmid containing *cat* gene flanked by FRT sites; Cm^R^ Ap^R^	Datsenko and Wanner (2000)
pKD46	Plasmid expressing γ, β, and *exo* recombination genes of phage λ under control of P_BAD_; temperature-sensitive replicon; Ap^R^	Datsenko and Wanner (2000)
pCP20	Plasmid expressing the FLP (flippase) gene, directing recombination of FRT sites; temperature-sensitive replicon; Ap^R^ Cm^R^	Datsenko and Wanner (2000)
pUC18	Cloning vector; Ap^R^	Laboratory collection
pUCGm*lox*	pUC18-based vector containing the *lox*-flanked *aacC1* gene; Ap^R^ Gm^R^	Quénée et al. (2005)
pSF100	pGP704 suicide plasmid; *pir* dependent; Ap^R^ Km^R^	Rubirés et al. (1997)
pCM157	*cre* expression vector; Tc^R^	Marx and Lidstrom (2002)

**Table 2:** Oligonucleotides used in this study.

Primer	Sequence (5′-3′)
Linker 1	TTTCTGCTCGAATTCAAGCTTCTAACGATGTACGGGGACACATG
Linker 2	TGTCCCCGTACATCGTTAGAACTACTCGTACCATCCACAT
Y linker primer	CTGCTCGAATTCAAGCTTCT
NK_Cm_DWN	CCTCCCAGAGCCTGATAA
EC_HycE_pKD3_1	GCCGTGCCGGTTTTGATGACTTTTTTGATAAAGGTAAACATGGCGATTCCATGGGAATTAGCCATGGTCC
EC_HycE_pKD3_2	TTTTTAGCGTTCGTCTCCTTGCTGGCGGCGTGATTAAAGAGAGTTTGAGCGTGTAGGCTGGAGCTGCTTC
SP_HycE_1(XbaI)	GCAGTCTAGAATCAGCGTCTGGTTCATTGG
SP_HycE_2(XbaI)	ACTCTCTAGATTATCTGTTCGCCGTGGTGC
SP_HycE_3(XhoI)	GCGACTCGAGCATGATGTTCCTACTTGTGAATTAGC
SP_HycE_4(XhoI)	GCACTCGAGCGGAAAAACGCACCGTTTTAA
LoxP_Gm_1(XhoI)	AACTCGAGCTTCAGCTGTACAATTGGTAC
LoxP_Gm_2(XhoI)	AACTCGAGACCGGTTAACACGCG

### SCREENING FOR MUTANTS THAT HAVE LOST STATIONARY-PHASE DEACIDIFICATION CAPACITY

A random knockout library of *E. coli* MG1655 containing pTrc99A-P_trc_-*budAB* was constructed using λNK1324, which carries a mini-Tn*10* transposon with a Cm resistance gene, according to the protocol described by Kleckner et al. (1991). The mutants were subsequently grown in 300 μl LB medium with glucose, IPTG, Ap, and Cm in a 96-well plate. The plates were sealed with an oxygen impermeable cover foil and incubated without shaking at 37°C. After 24 h, medium acidification was analyzed by adding 5 μl of a 0.06% w/v methyl red solution in 60% v/v ethanol to 200 μl culture (MR test). For mutants that no longer deacidified the medium, the remaining 100 μl culture was subjected to the Voges–Proskauer (VP) test by adding 30 μl of 5% w/v α-naphthol and 10 μl of 40% w/v KOH to 100 μl of culture. To quantify acetoin production, the mixture was stirred vigorously after 1 h and the optical density at 550 nm (OD_550_) was measured. Acetoin concentrations were determined using a standard curve relating the OD_550_ with the acetoin concentration in LB medium. From mutants that did not deacidify culture medium and still produced acetoin, transposon insertion sites were determined using the method described by Kwon and Ricke (2000). Briefly, genomic DNA of the mutants was isolated, digested with NlaIII and ligated with a Y-shaped linker, composed of oligonucleotides linker 1 and linker 2. Next, a PCR amplification was carried out using a transposon-specific primer (NK_Cm_DWN) and a primer specific to the Y-shaped linker (Y linker primer). The PCR product was subsequently sequenced using the transposon-specific primer and the insertion site was determined based on the known genome sequence of *E. coli* MG1655.

### CONSTRUCTION OF *hycE* MUTANTS IN *E. coli* AND *S. plymuthica*

The deletion of *hycE* in *E. coli* MG1655 was achieved using the lambda red recombinase system described by Datsenko and Wanner (2000), followed by removal of the introduced antibiotic resistance cassette using the FRT/FLP recombination system. Briefly, 70-bp PCR primers were designed comprising a 50-bp 5′ part complementary to the region down- or upstream of *hycE* and a 20-bp 3′ part allowing amplification of the FRT-flanked Cm resistance cassette present in the plasmid pKD3. The purified PCR product was electrotransformed into *E. coli* MG1655 containing the pKD46 plasmid providing the lambda red recombinase. The resistance cassette was subsequently removed by expression of the flippase recombination enzyme (FLP) of the FRT/FLP recombination system on the temperature-sensitive pCP20 plasmid.

To delete the *hycE* gene in *S. plymuthica* RVH1, a fragment encompassing 643 bp upstream and 559 bp downstream of the gene was PCR-amplified using primers SP_HycE_1(XbaI) and SP_HycE_2(XbaI), cut with XbaI, ligated into a XbaI-digested pUC18 vector and transformed into *E. coli* DH5α. The resulting plasmid pUC18-*hycE* was used as a template for PCR using the outward-oriented primers SP_HycE_3(XhoI) and SP_HycE_4(XhoI). In a separate reaction, the *loxP* flanked Gm resistance cassette from plasmid pUCGm*lox* was amplified using primers LoxP_Gm_1(XhoI) and LoxP_Gm_2(XhoI). Both PCR products were then cleaved with XhoI and ligated together, generating pUC18-*hycE::aacC1*, which was transformed in *E. coli* DH5α. The *hycE::aacC1* insert from this plasmid was then amplified using primers SP_HycE_1(XbaI) and SP_HycE_2(XbaI), cut with XbaI, ligated into a XbaI-digested pSF100 vector and transformed into *E. coli* S17-1 λ*pir*. After conjugation of the resulting plasmid pSF100-*hycE::aacC1* into *S. plymuthica* RVH1 (which does not support replication of this suicide plasmid), transconjugants were selected on LB agar with Gm at 15°C. This temperature allows good growth of *S. plymuthica* but prevents growth of *E. coli* S17-1 λ*pir*. Loss of Km resistance (pSF100 marker) was assessed by replica plating on LB agar with Km. The Gm resistance cassette was then spliced out using the *cre* recombinase on plasmid pCM157, which catalyzes site specific recombination between *loxP* sites. Restriction endonucleases and T4 DNA ligase were purchased from Thermo Scientific (St. Leon Rot, Germany) and used according to the supplier’s instructions.

### CHARACTERIZATION OF FERMENTATIVE GROWTH AND FERMENTATION END PRODUCTS

Strains were first grown overnight at the appropriate incubation temperature in 4 ml LB. For strains containing pTrc99A or pTrc99A-P_trc_-*budAB*, Ap was added to ensure plasmid maintenance. Since *S. plymuthica* RVH1 is somewhat Ap resistant, Cb was used instead of Ap. Next, the cultures were diluted 1:1000 in tubes containing 30 ml LB with glucose and, when appropriate, IPTG and Ap or Cb. Five ml of paraffin oil was layered on top of the cultures to create anaerobic conditions and the tubes were incubated at the appropriate incubation temperature for 48 h. The cultures were sampled at regular time points to determine cell concentrations, medium pH and acetoin concentration, and for analysis of fermentation end products. Plate counts were determined by spot-plating (5 μl) a decimal dilution series in potassium phosphate buffer (10 mM; pH 7.00) on LB agar. Gas production was evaluated qualitatively using Durham tubes. Fermentation end products were analyzed in 600 μl culture supernatants stored at –20°C. Succinic, lactic, formic, and acetic acid, ethanol, and glucose were determined via high-performance liquid chromatography (HPLC; Agilent 1200 series) using an ion exclusion column (Aminex® HPX-87H) maintained at 55°C, and with 5 mM H_2_SO_4_ as the mobile phase (0.6 ml/min). The system was equipped with a refractive index detector operating at 35°C and a diode array detector set at 210 nm.

### STATISTICAL ANALYSIS

All experiments were carried out in triplicate using independent cultures, and results are presented as the mean values ± SD. Statistical significance between mean values were determined by Student’s *t*-test analysis using the Microsoft Excel statistical package. Results were reported as significant when a *p*-value of <0.05 was obtained, based on a two-sided *t*-test with unequal variance.

## RESULTS AND DISCUSSION

### INTRODUCTION OF ACETOIN SYNTHESIS PATHWAY IN MIXED-ACID FERMENTERS

**FIGURE 1:**
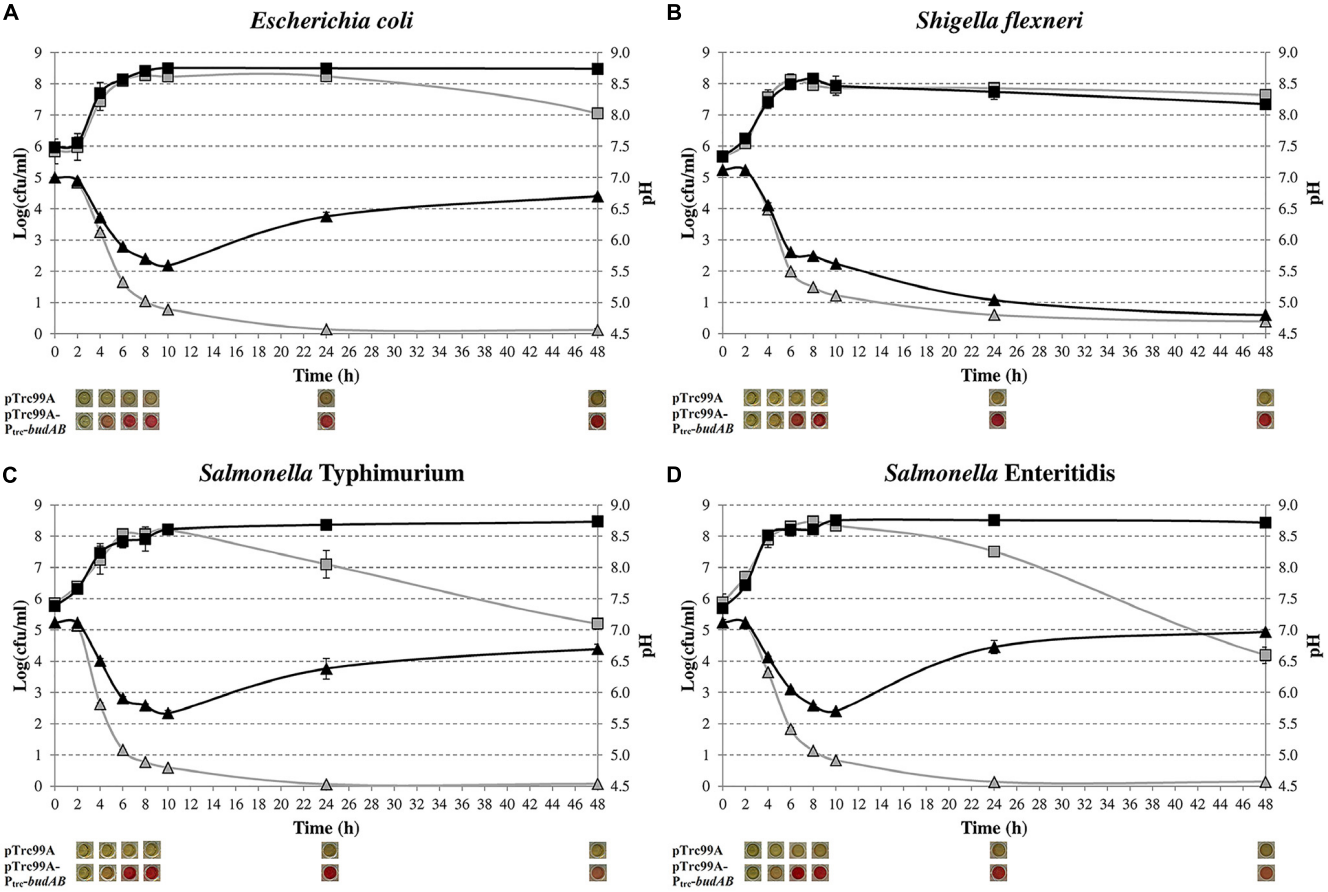
**Growth of *Escherichia coli* MG1655 **(A)**, *S. flexneri* ATCC 12022 **(B)**, *S.* Typhimurium LT2 **(C)** and *S.* Enteritidis ATCC 13076 **(D)** in LB medium containing 5 g/l glucose, 1 mM IPTG and 100 μg/ml ampicillin (Ap), in sealed microtiter plates incubated at 37°C for 48 h.** Cell numbers (squares) and medium pH (triangles) of strains harboring pTrc99A (gray) or pTrc99A-P_trc_-*budAB* (black) are shown. Pictures below the figures show the results of the VP test, with a red color indicating the presence of acetoin. Error bars represent SD.

Previously, we introduced the *budAB* operon from *S. plymuthica* RVH1, encoding the α-ALS and α-ALD of the acetoin synthesis pathway, in *E. coli* MG1655 and observed that this attenuated lethal medium acidification during fermentative growth on glucose (Vivijs et al., 2014a). Here, we extended this experiment to *S.* Typhimurium, *S.* Enteritidis, and *S. flexneri* by introducing the pTrc99A-P_trc_-*budAB* plasmid into these organisms to see whether other mixed-acid fermenters would show a similar behavior. **Figure 1** shows the growth curves and medium pH during fermentative growth in glucose-containing LB medium of these bacteria with and without the *budAB* genes. As expected, *E. coli*, both *Salmonella* strains and *S. flexneri* without *budAB* strongly acidified the medium (to pH 4.50–4.70 after 48 h) and this resulted in cell death during the stationary phase. Introduction of the *budAB* genes did not change growth of the bacteria until stationary phase was reached, but it changed the pH profile of the *E. coli* and *Salmonella* cultures in two aspects. Firstly, the acidification during the growth phase was less strong, reaching a minimum pH of about 5.60. Secondly, the pH increased again during stationary phase, up to 6.60–7.00 after 48 h. As a result, plate counts remained almost constant once they had reached their maximal stationary phase level (10–48 h).

Surprisingly, a different pattern was observed in *S. flexneri*. Introduction of the *budAB* genes also attenuated medium acidification during the growth phase (pH 5.60 after 10 h), but no deacidification occurred during stationary phase. As a result, this culture reached a final pH of 4.80 after 48 h and the plate counts decreased to a similar extent as those of the strain without *budAB* genes. The strain with the *budAB* genes produced acetoin in similar amounts as the *E. coli* and *Salmonella* strains carrying these genes, so that poor expression of the acetoin pathway could be ruled out to explain the different behavior of *S. flexneri.* Therefore, proton consumption in the acetoin production pathway cannot fully explain the deacidification during stationary phase in *E. coli* and *Salmonella,* and it can be concluded that other deacidification mechanisms must be involved.

### SCREENING FOR LOSS OF DEACIDIFICATION CAPACITY IN *E. coli* CONTAINING A FUNCTIONAL ACETOIN PATHWAY

In order to identify additional mechanisms involved in stationary-phase deacidification, we performed random transposon mutagenesis in *E. coli* MG1655 containing the pTrc99A-P_trc_-*budAB* plasmid and searched for mutants that were unaffected in acetoin production (VP test), yet were no longer able to increase the pH of glucose-containing LB medium at 37°C after 24 h (MR test), thus having a MR+/VP+ phenotype. Although in most *Enterobacteriaceae* a positive VP test is usually associated with a negative MR test (MR–/VP+, e.g., *Enterobacter aerogenes*) and vice versa (MR+/VP–, e.g., *E. coli*), there are also some species in this family (e.g., *Enterobacter intermedius*, *Klebsiella planticola*, or *Serratia liquefaciens*) reported to be positive for both tests (MR+/VP+; Holt et al., 1994).

**FIGURE 2:**
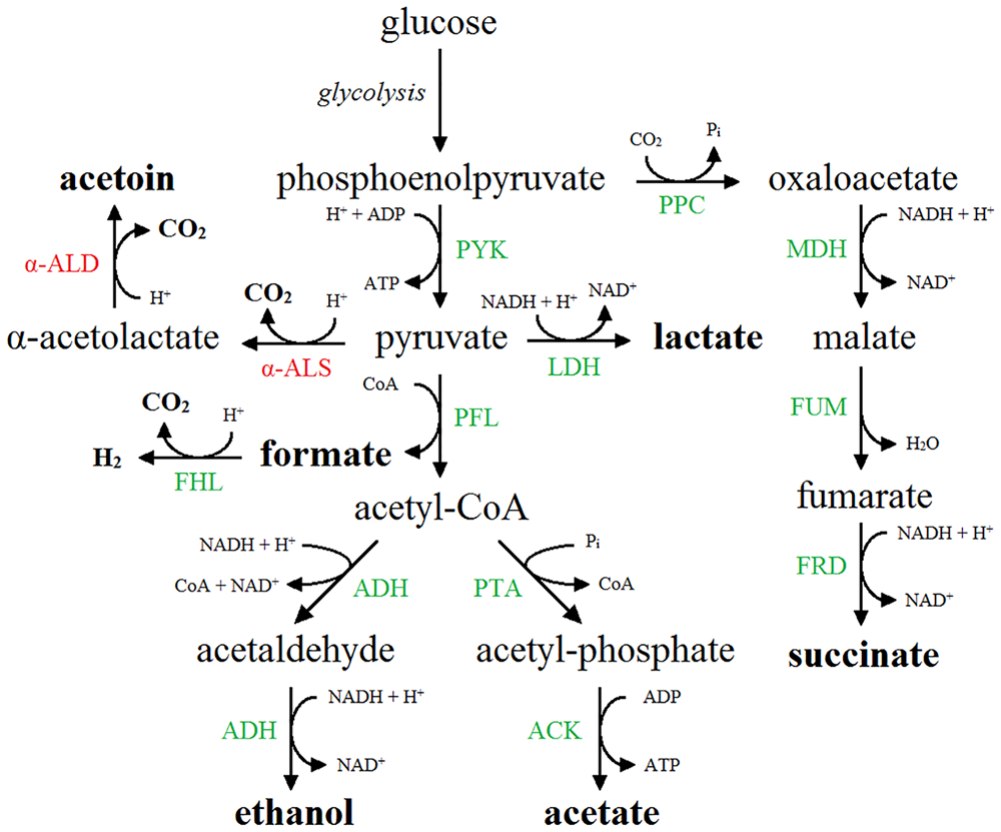
**Mixed-acid fermentation pathway in *E. coli* expressing the acetoin synthesis operon (*budAB* operon).** End products are shown in boldface. Native enzymes are shown in green. The enzymes of the additional acetoin synthesis pathway, in which pyruvate is converted to α-acetolactate by α-ALS (BudB) and further to acetoin by α-ALD (BudA), are shown in red. α-ALS, α-acetolactate synthase; α-ALD, α-acetolactate decarboxylase; ACK, acetate kinase; ADH, acetaldehyde dehydrogenase/alcohol dehydrogenase; FHL, formate hydrogen lyase complex; FRD, fumarate reductase; FUM, fumarase; LDH, lactate dehydrogenase; MDH, malate dehydrogenase; PFL, pyruvate formate lyase; PPC, phosphoenolpyruvate carboxylase; PTA, phosphate acetyltransferase; PYK, pyruvate kinase.

Out of 6.048 mutants screened, 34 MR+/VP+ mutants were identified and their phenotype was confirmed after transferring the mutation to a native MG1655 strain by P1-transduction, followed by transformation of pTrc99A-P_trc_-*budAB*. Identification of the transposon insertion sites of these 34 mutants led to 16 different genes (**Table 3**). Interestingly, all genes were related to the metabolism of formate, one of the acids formed by mixed-acid fermentation. Formate is produced by the pyruvate formate lyase (PFL) enzyme, which catalyzes the CoA-dependent cleavage of pyruvate to formate and acetyl-CoA (Sawers and Böck, 1988). An overview of the fermentation routes present in *E. coli* containing pTrc99A-P_trc_-*budAB* is shown in **Figure 2**. The formate that is produced and secreted can also be reimported in the cell through the FocA channel and become disproportionated to CO_2_ and H_2_ by the membrane-associated FHL complex (Sawers, 2005; Lü et al., 2012; Beyer et al., 2013). This complex consists of the formate dehydrogenase H (FDH-H), a selenoprotein carrying a molybdenum cofactor, and hydrogenase 3, a nickel-containing protein complex (Bagramyan and Trchounian, 2003). FDH-H catalyzes the oxidation of formate (HCOO^-^), generating CO_2_ and H^+^. The electrons from this reaction are transferred via several subunits of the FHL complex to hydrogenase 3, where they combine with two cytoplasmic protons to form dihydrogen. This pathway is thus a net consumer of protons and is used by *E. coli* to counteract acidification (Leonhartsberger et al., 2002). All gene products found in our screening could be linked to this particular pathway: FdhF (FDH-H), HycB, HycD, and HycE are part of the FHL complex (Bagramyan and Trchounian, 2003); HycI and HypE are both involved in maturation of the large subunit of hydrogenase 3 (Forzi and Sawers, 2007); SelA and SelD take part in the biosynthesis of selenocysteine, and mutants lacking these gene products fail to synthesize FDH-H (Leinfelder et al., 1988; Driscoll and Copeland, 2003); ModC is the ATP binding subunit of the molybdate ABC transporter and MoeA, MoeB, and Mog are other ancillary enzymes that participate in the biosynthesis of the molybdenum cofactor (Sawers, 1994; Grunden and Shanmugam, 1997; Leimkühler et al., 2001; Nichols and Rajagopalan, 2002); FdhD is an accessory protein functioning as a sulfurtransferase between IscS and FdhF and is required for FDH activity (Thomé et al., 2012); FocA and PflB are coexpressed from a single operon and form a bidirectional formate channel and the PFL enzyme, respectively (Lü et al., 2012); FhlA, finally, is a transcriptional activator of the FHL system (Leonhartsberger et al., 2002). In conclusion, the mutant screening approach provides a strong indication that the disproportionation of formate is responsible for the stationary-phase deacidification capacity in *E. coli* containing the *budAB* genes.

**Table 3:** List of genes knocked out in transposon insertion mutants of *E. coli* MG1655 containing pTrc99A-P_**trc**_-*budAB* that had lost the stationary-phase deacidification capacity but still produced acetoin (MR+/VP+).

Gene	Description
*fdhD*	Redox enzyme maturation protein (REMP) for FdnG/FdoG; required as a sulfurtransferase for FDH activity
*fdhF*	FDH-H
*fhlA*	FHL system activator
*focA*	Formate channel
*hycB*	FHL complex iron–sulfur protein
*hycD*	FHL complex inner membrane protein
*hycE*	Hydrogenase 3 large subunit
*hycI*	Maturation endoprotease for hydrogenase 3 large subunit HycE
*hypE*	Maturation protein required for the assembly of the CN ligand of the NiFe metal center of hydrogenase 1, 2, and 3.
*modC*	ATP binding subunit of the molybdate ABC transporter
*moeA*	Molybdopterin molybdenumtransferase
*moeB*	Molybdopterin-synthase adenylyltransferase
*mog*	Molybdochelatase incorporating molybdenum into molybdopterin
*pflB*	Pyruvate formate lyase
*selA*	Selenocysteine synthase
*selD*	Selenophosphate synthase

In addition to hydrogenase 3, *E. coli* also possesses three other hydrogenases catalyzing the reversible reaction 2H^+^ + 2e^-^ ↔ H_2_. Hydrogenase 1 and 2 are H_2_-oxidizing enzymes which are maximally induced at low and alkaline pH, respectively (Trchounian et al., 2012). Hydrogenase 4 is not well characterized and its subunits have not been isolated and studied yet, but it may be part of a second FHL complex that may produce H_2_ at neutral and slightly alkaline pH (Self et al., 2004; Trchounian and Sawers, 2014). The contribution of hydrogenase 3 to acid resistance has been demonstrated previously since anaerobic cultures of *E. coli* W3110 Δ*hycE* showed a 20-fold loss in survival of an extreme acid stress (2 h at pH 2.0) when compared to the wild-type strain (Noguchi et al., 2010). This finding suggested that the FHL complex supports survival of extreme acid challenge by counteracting intracellular acidification. Our results now show that the complex can also accomplish an increase of the environmental pH during growth under moderate acid stress, thereby preventing stationary phase cell death during fermentative growth.

The observation that acetoin-producing *S. flexneri* showed reduced acidification in the exponential growth phase, but did not deacidify the medium during the stationary phase (**Figure 1**), can also be linked to formate conversion. Although *S. flexneri* closely resembles *E. coli* at the genetic level, *Shigella* species (with the exception of a few strains) do not produce gas during carbohydrate fermentation (Brenner et al., 1982; Germani and Sansonetti, 2006). We confirmed that the *S. flexneri* strain used in this study did not produce gas from glucose and the absence of this mechanism may thus explain our observation. The reason why *Shigella* species do not produce gas in the presence of glucose is unclear. The genes encoding the FDH-H and the hydrogenase 3 are present in the *Shigella* genome, but apparently no functional FHL complex is formed.

### EFFECT OF HYDROGENASE 3 INACTIVATION ON FERMENTATIVE GROWTH OF ACETOIN-PRODUCING *E. coli*

**FIGURE 3:**
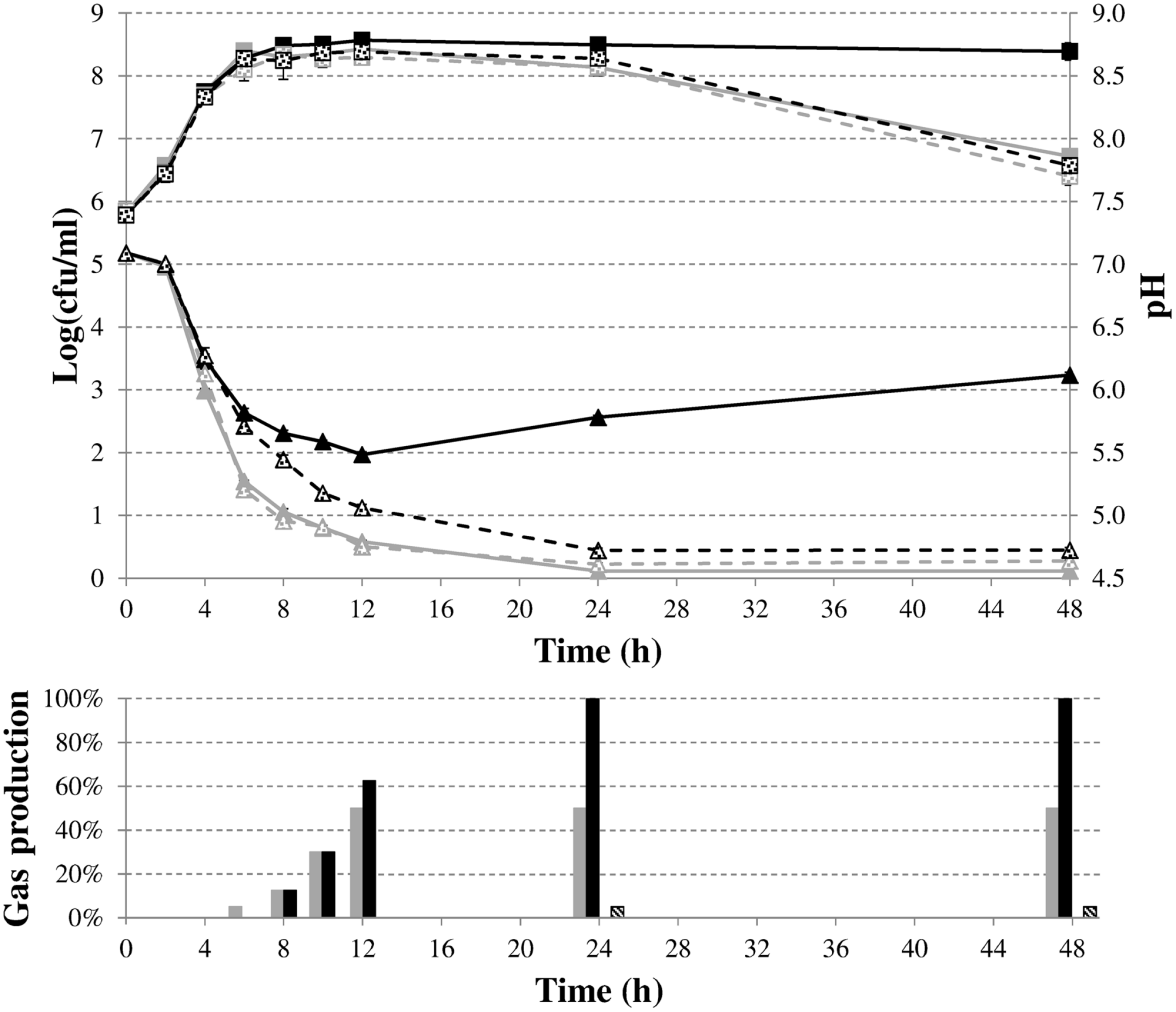
**Cell numbers (squares) and medium pH (triangles) during fermentative growth of *E. coli* MG1655 wild-type (solid lines) or Δ*hycE* (dashed lines) containing pTrc99A (gray) or pTrc99A-P_**trc**_-*budAB* (black) in LB medium with 5 g/l glucose, 1 mM IPTG and 100 μg/ml Ap at 37°C for 48 h.** Error bars represent SD. Gas production (expressed as % of the volume of the Durham tube) is shown at the bottom.

To characterize in more detail the role of formate disproportionation on the capacity of *E. coli* (with or without *budAB* genes) to attenuate medium acidification during fermentative growth, we constructed a clean deletion of the *hycE* gene. Since this gene encodes the large subunit of the hydrogenase 3 that contains the active site for proton reduction to dihydrogen (Trchounian et al., 2012), its deletion completely blocks the conversion of formate to CO_2_ and H_2_. Next, *budAB*-less and *budAB*-containing wild-type and Δ*hycE* strains of *E. coli* MG1655 were grown for 48 h in glucose-containing LB medium sealed from the air with a paraffin oil layer and with a Durham tube to observe gas production. Plate counts, medium pH, gas production and acetoin concentrations were determined at regular time points (**Figure 3**; **Table 4**). Knockout of *hycE* did not have any effect on the pH profile during fermentative growth of *budAB*-less *E. coli* over the entire 48 h growth period. In the *budAB*-containing strains, the effect of *hycE* deletion depended on the growth phase. During the exponential phase (first 6 h), *hycE* deletion had no effect on the acidification, but it can be seen that the acidification was slightly less compared to the two *budAB*-less strains, in line with the earlier observations shown in **Figure 1**. However, from the onset of stationary phase, the pH profile of both acetoin-producing strains diverged strongly. While acidification by the *budAB*-containing wild-type strain slowed down and reversed into deacidification after 12 h of growth (as already shown in **Figure 1**), acidification by the *budAB*-containing Δ*hycE* mutant was sustained until 24 h, after which the pH remained stable at a low value (pH = 4.72). Since both strains produced similar amounts of acetoin, and acetoin production stopped after 10 h (**Table 4**), it can be concluded that the stationary-phase deacidification by the *budAB*-containing wild-type *E. coli* MG1655 is not a direct consequence of proton consumption during acetoin production.

**Table 4:** Acetoin production (in mM) by *E. coli* MG1655 containing pTrc99A-P_**trc**_-*budAB* or *E. coli* MG1655 Δ*hycE* containing pTrc99A-P_**trc**_-*budAB* during fermentative growth in LB with 5 g/l glucose, 1 mM IPTG, and 100 μg/ml ampicillin (Ap) for 48 h.

Time (h)	*E. coli* MG1655 pTrc99A-P_trc_-*budAB*	*E. coli* MG1655 Δ*hycE* pTrc99A-P_trc_-*budAB*
4	0.7 ± 0.2	0.5 ± 0.2
6	5.3 ± 0.7	5.0 ± 0.4
8	9.8 ± 0.9	7.1 ± 1.3
10	21.8 ± 0.8	18.4 ± 0.5
12	19.4 ± 1.2	12.5 ± 1.1
24	18.7 ± 0.7	12.3 ± 0.5
48	20.5 ± 2.3	11.1 ± 2.3

More likely, deacidification is triggered by proton consumption in the reaction carried out by the FHL complex since deletion of *hycE* resulted in loss of deacidification. This explanation is also supported by the observed gas production. Since CO_2_ is very soluble in water, gas accumulation in a Durham tube can be mainly ascribed to H_2_ production, and is thus indicative of the action of the FHL complex (White, 2000). Both strains with an intact FHL complex produced more or less the same amount of gas at 12 h, filling approximately half of the Durham tube with gas (**Figure 3**). However, no additional gas production was seen in case of wild-type *E. coli* after 12 h, while the Durham tubes in case of acetoin-producing *E. coli* were completely filled with gas after 24 h, and additional gas bubbles were formed in the medium after 48 h. On the other hand, the Δ*hycE* mutant did not produce any gas, while only a small amount of gas was observed in the *budAB*-containing Δ*hycE* mutant, which might be the result of CO_2_ production during acetoin formation (**Figure 3**).

The evolution of plate counts during stationary phase in this experiment was generally in line with the observed pH changes, with cell death taking place in the strongly acidified cultures. In particular, lethal acidification could not be prevented by acetoin fermentation in a *budAB*-containing Δ*hycE* mutant since plate counts of this strain significantly decreased after the stationary phase, as was also the case for the two *budAB*-less strains performing a mixed-acid fermentation. Cell death can be explained by the combination of the low pH environment and the toxic accumulation of organic acids.

### ANALYSIS OF METABOLITES PRODUCED DURING FERMENTATIVE GROWTH OF *E. coli*

**FIGURE 4:**
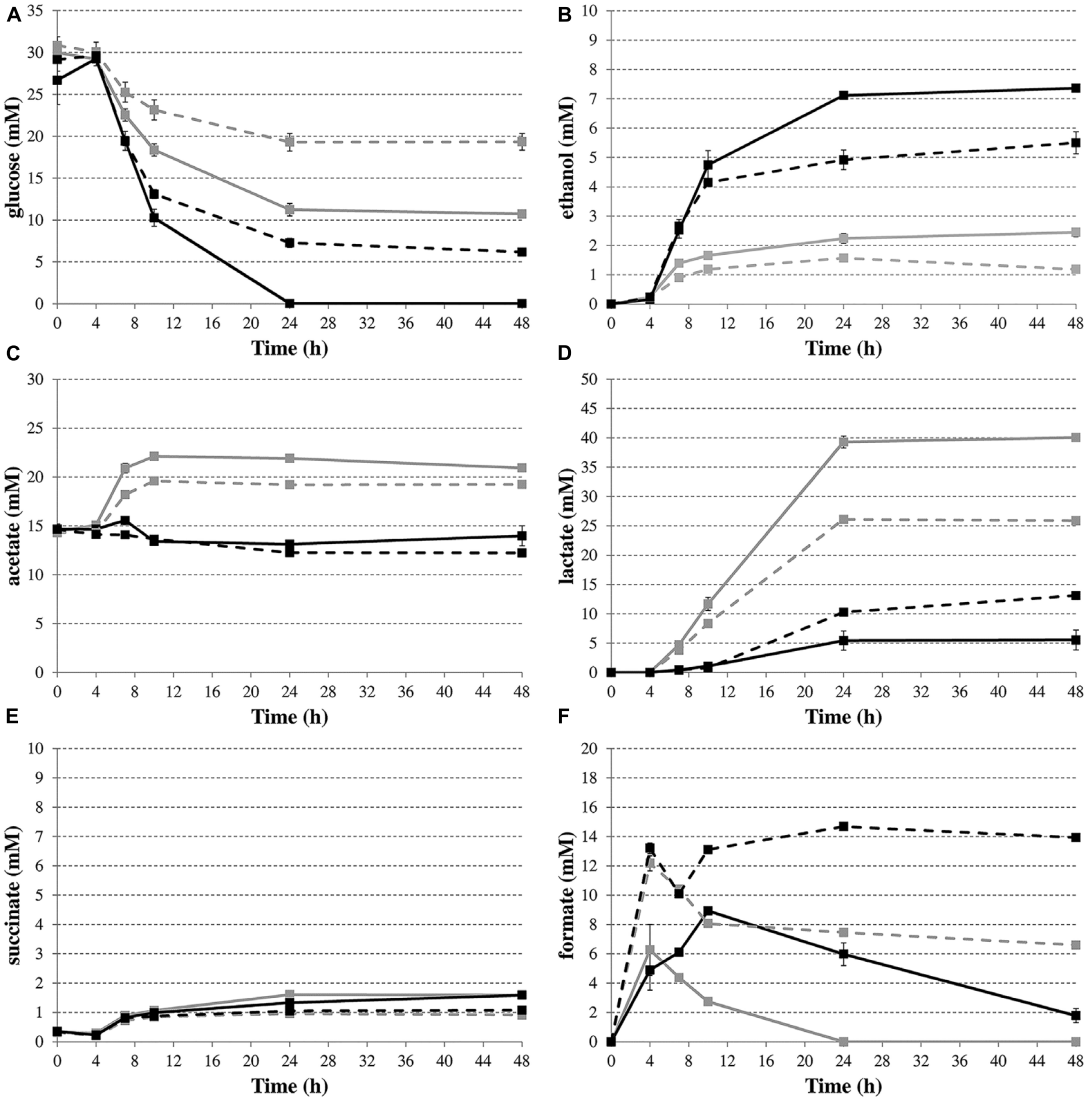
**Time profiles of glucose consumption **(A)** and production of the metabolites ethanol **(B)**, acetate **(C)**, lactate **(D)**, succinate **(E)**, and formate **(F)** during fermentative growth of *E. coli* MG1655 wild-type (solid lines) or Δ*hycE* (dashed lines) containing pTrc99A (gray) or pTrc99A-P_**trc**_-*budAB* (black) in LB medium with 5 g/l glucose, 1 mM IPTG and 100 μg/ml Ap at 37°C for 48 h.** Error bars represent SD.

To provide more direct evidence for the involvement of formate disproportionation in the deacidification capacity of *budAB*-containing *E. coli*, glucose consumption and the production of metabolites were determined by HPLC during fermentative growth in LB with glucose (**Figure 4**). Succinate concentrations (**Figure 4E**) remained low for all strains during the course of the experiment. On the other hand, the *budAB* genes caused a marked shift in the production of two of the major acids of the mixed-acid fermentation pathway, especially in the late exponential and stationary growth phase, with no more acetate and much less lactate being produced (**Figures 4C,D**, respectively). With regard to formate (**Figure 4F**), the highest formate accumulation was seen in the Δ*hycE* mutants, probably because these have lost their major route to convert formate to H_2_ and CO_2_. During the stationary growth phase (up to 48 h), the formate concentrations remained almost constant in the *hycE^-^* strains, but strongly decreased in the *hycE*^+^ strains, indicating the reuptake and conversion of formate to CO_2_ and H_2_. Interestingly, a close look at the formate accumulation curves of the Δ*hycE* mutants reveals a transient decline in the late exponential growth phase (onset at 4 h of growth). Also in the *hycE*^+^ background a decline (*budAB*-less strain) or a diminished accumulation (*budAB*-containing strain) of formate was observed in this phase. A possible explanation for this is the activity of the FDH-N, which also catalyzes the oxidation of formate to CO_2_ (Sawers, 1994). However, FDH-N transfers the electrons to nitrate (via a nitrate reductase) instead of protons and has a much higher affinity for formate than the FDH-H (Leonhartsberger et al., 2002), which could explain why it is active in an earlier growth stage. The activity of FDH-N is limited, however, because LB medium contains only a small amount of nitrate. The disproportionation of formate (**Figure 4F**) by the *hycE*^+^ strains lasted longer when the *budAB* genes were present (48 h) than when they were absent (24 h), probably because a higher amount of formate was produced. This was also reflected by an increased gas production in the presence of the *budAB* genes during this phase, as reported above (**Figure 3**). Finally, ethanol was produced in higher quantities by the *budAB*-containing strains (**Figure 4B**).

As a final experiment to demonstrate that formate conversion causes medium deacidification during stationary phase, 5 or 10 mM formate from a 1 M solution (pH 5.50) was added to the medium after 10 h of fermentative growth of *budAB*-containing *E. coli* MG1655 and the pH was subsequently measured after 10, 24, and 48 h. As expected, the addition of formate in the medium resulted in a significantly stronger pH increase during stationary phase (**Table 5**).

**Table 5:** Effect of exogenous formate addition on pH of fermentation medium.

Concentration of formate added at 10 h	10 h	24 h	48 h
0 mM	5.64 ± 0.03^a^	5.81 ± 0.04^a^	6.14 ± 0.02^a^
5 mM	5.66 ± 0.05^a^	5.96 ± 0.04^b^	6.35 ± 0.04^b^
10 mM	5.63 ± 0.08^a^	6.05 ± 0.04^c^	6.48 ± 0.05^c^

**E. coli* MG1655 harboring pTrc99A-P_trc_-*budAB* was grown in LB medium with 5 g/l glucose, 1 mM IPTG, and 100 μg/ml Ap. Formate was added at 10 h and pH was measured at 10, 24, and 48 h. ^a-c^ pH values with different superscripts in the same column are significantly different (*p* < 0.05)*.

Taken together, the metabolite profiles lead us to propose the following model to explain the effect of introduction of the *budAB* genes in *E. coli* (see **Figure 2**). The introduction of these genes diverts part of the pyruvate generated from glycolysis to acetoin production. At the same time, possibly because of a reduced cellular pyruvate pool, the balance between the mixed-acid fermentation routes is shifted, with lactate production being almost shut down. Nevertheless, since the *budAB*-containing strain produced higher amounts of formate (see previous paragraph), it maintains a higher flux of pyruvate to acetyl-CoA, as also indicated by the higher glucose consumption. This can be explained by the reduced acid production and consequently the reduced metabolic inhibition. The fate of acetyl-CoA is also different in the *budAB-*containing strain. This is necessarily so, because the reduced production of lactic acid creates an excess of NADH that must be reoxidized by another route to maintain the cellular redox balance. As can be seen in **Figure 2**, this is only possible by increasing ethanol production at the expense of acetate production. This is indeed what happens, since the *budAB-*containing strain no longer produces acetate and has increased ethanol production. Since acetate production is coupled to the generation of an extra ATP, introduction of the acetoin pathway reduces the ATP yield per mole of glucose fermented. However, this does not result in reduced growth rate (**Figures 1** and **3**), because it is compensated by a higher glucose turnover. Thus, although the total biomass production (maximal cell density reached in early stationary phase) is approximately the same for all the strains, the *budAB*-containing strains require much more glucose to achieve this (**Figure 4A**).

### ROLE OF HYDROGENASE 3 IN FERMENTATIVE GROWTH OF *S. plymuthica* RVH1

**FIGURE 5:**
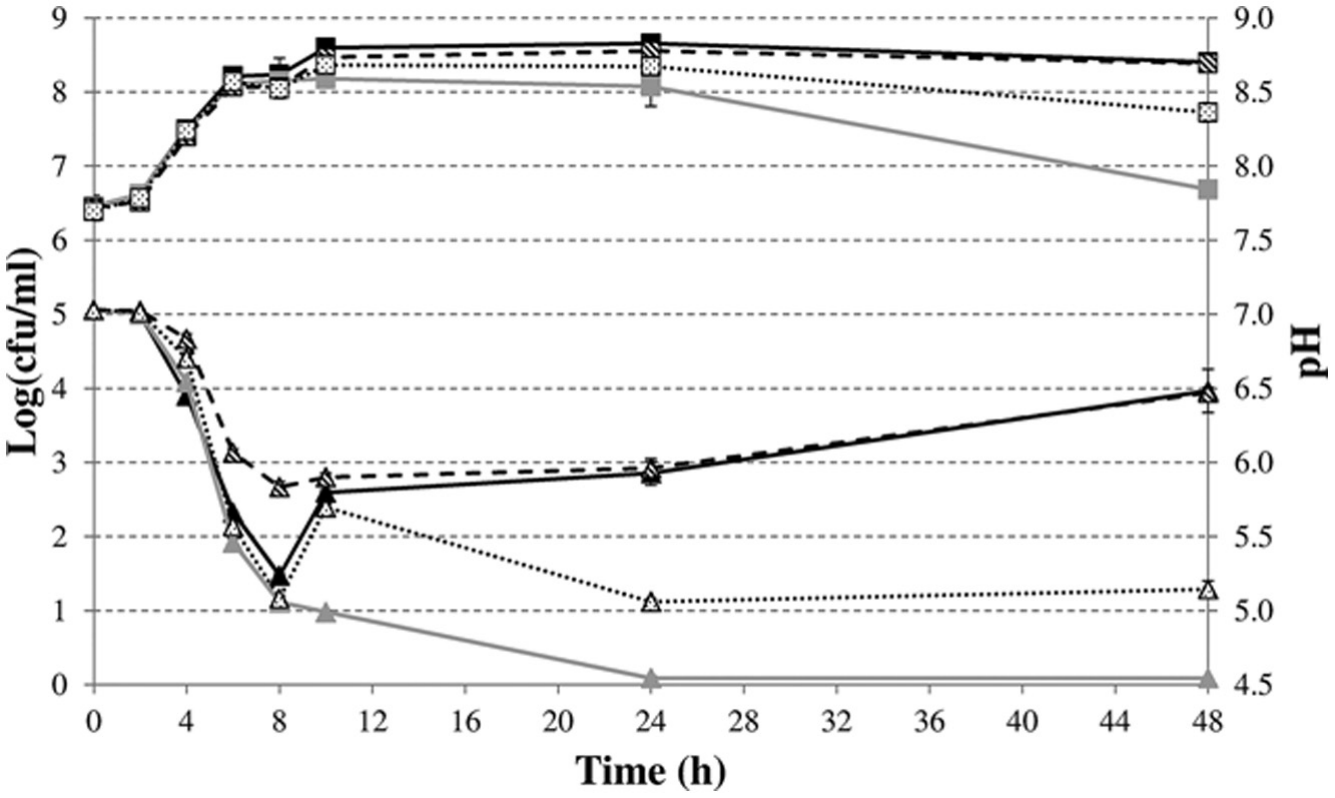
**Cell numbers (squares) and medium pH (triangles) during fermentative growth of *S. plymuthica* RVH1 wild-type (black solid lines), *budAB::cat* (gray solid lines), *budAB::cat* containing pTrc99A-P_**trc**_-*budAB* (dashed line), or Δ*hycE* (dotted line) in LB medium with 5 g/l glucose at 30°C for 48 h.** For the strain containing pTrc99A-P_trc_-*budAB*, 1 mM IPTG and 200 μg/ml carbenicillin (Cb) were added to the medium. Error bars represent SD.

Finally, we investigated whether the FHL complex also attenuates acid formation and drives deacidification during fermentative growth of a natural 2,3-butanediol fermenter, using *S. plymuthica* RVH1 as a model. To this end, we constructed a Δ*hycE* mutant in this strain. The evolution of medium pH for *S. plymuthica* RVH1 wild-type shows three phases (**Figure 5**). There was a decrease during the first 8 h, followed by a rapid increase between 8 and 10 h, and then a slower increase until 48 h. The initial pH increase is probably due to the switch to 2,3-butanediol production in the late exponential phase since it was lost upon knockout of the 2,3-butanediol pathway (*budAB::cat*) but not by knockout of hydrogenase 3 (Δ*hycE*). In contrast, the deacidification during stationary phase required both an active 2,3-butanediol pathway and an active hydrogenase 3. Genetic complementation of the *budAB* mutant restored its pH profile to that of the wild-type strain. However, since this complemented strain produces acetoin under the control of the plasmid P_trc_ promoter right from the start of the experiment, its acidification is more attenuated than in the wild-type strain. Cell numbers declined after 48 h in the mutant strains that had lost or reduced deacidification capacity and, as a result, there was a clear correlation between cell numbers and medium pH at the end of the experiment.

Previously, the pH profile during glucose fermentation in the 2,3-butanediol fermenter *Enterobacter aerogenes* was divided into three phases (Johansen et al., 1975). The first phase was characterized by a rapid drop to about pH 5.8, in the second phase the pH remained almost constant at pH 5.6 and in the third phase the pH increased again to about 6.5. However, during the last phase, the total amount of acetoin and 2,3-butanediol remained constant and 2,3-butanediol was reoxidized to acetoin, indicating that the 2,3-butanediol pathway is not involved in this deacidification (Johansen et al., 1975). Our results demonstrate that –at least in *S. plymuthica*- the FHL complex is responsible for stationary-phase deacidification since the final pH was about 1.3 pH units lower in a *S. plymuthica* RVH1 Δ*hycE* mutant compared to the wild-type.

## Conflict of Interest Statement

The authors declare that the research was conducted in the absence of any commercial or financial relationships that could be construed as a potential conflict of interest.
